# Optimized Protocol for OnGuard2 Software in Studying Guard Cell Membrane Transport and Stomatal Physiology

**DOI:** 10.3389/fpls.2020.00131

**Published:** 2020-02-21

**Authors:** Sehar Shafaque, Yue Ma, Mengmeng Rui, Bingqing He, Ziyi Zhu, Fangbing Cao, Feibo Wu, Yizhou Wang

**Affiliations:** ^1^ Institute of Crop Science, College of Agriculture and Biotechnology, Zijingang Campus, Zhejiang University, Hangzhou, China; ^2^ Zhejiang Province Key Laboratory of Crop Germplasm, Zhejiang University, Hangzhou, China; ^3^ Jiangsu Co-Innovation Center for Modern Production Technology of Grain Crops, Yangzhou University, Yangzhou, China

**Keywords:** OnGuard model, system biology, stomatal behaviors, cellular homeostasis, membrane transport

## Abstract

Stomata are key innovation in plants that drives the global carbon and water cycle. In the past few decades, many stomatal models have been developed for studying gas exchange, photosynthesis, and transpirational characteristics of plants, but they provide limited information on stomatal mechanisms at the molecular and cellular levels. Quantitative mathematical modeling offers an effective *in silico* approach to explore the link between microscopic transporter functioning and the macroscopic stomatal characteristics. As a first step, a dynamic system model based on the guard cell membrane transport system was developed and encoded in the OnGuard software. This software has already generated a wealth of testable predictions and outcomes sufficient to guide phenotypic and mutational studies. It has a user-friendly interface, which can be easily accessed by researchers to manipulate the key elements and parameters in the system for guard cell simulation in plants. To promote the adoption of this OnGuard application, here we outline a standard protocol that will enable users with experience in basic plant physiology, cell biology, and membrane transport to advance quickly in learning to use it.

## Introduction

Stomata are small pores in the leaf epidermis of plants providing the major pathway for gas exchange (Hetherington and Woodward, 2003). They are regulated by pairs of guard cells to balance the demands for CO_2_ in photosynthesis against foliar water loss *via* transpiration. There is no doubt that stomata play a very important role in the carbon and water budgets of plants (Lawson and Blatt, 2014; Blatt et al., 2017). A better understanding of stomatal functioning is vital for improving the water use efficiency in agriculture worldwide. Over the last few decades, many stomatal models (Damour et al., 2010; Buckley and Mott, 2013; Buckley, 2017; Franks et al., 2018), especially stomatal conductance models, were proposed to investigate and predict plant-environment interactions in response to the ongoing global climate change. Most of them are empirical or phenomenological models focusing on a limited number of environmental factors that affect stomatal movement. Nonetheless, these models have been successfully applied in the prediction of gas exchange in how plants respond to environmental changes. However, the lack of essential “macro-micro” connections to molecular and cellular mechanics hinders the insightful understanding of stomatal regulation and its broader agricultural, plant biological, and ecological applications.

**Figure 1:**
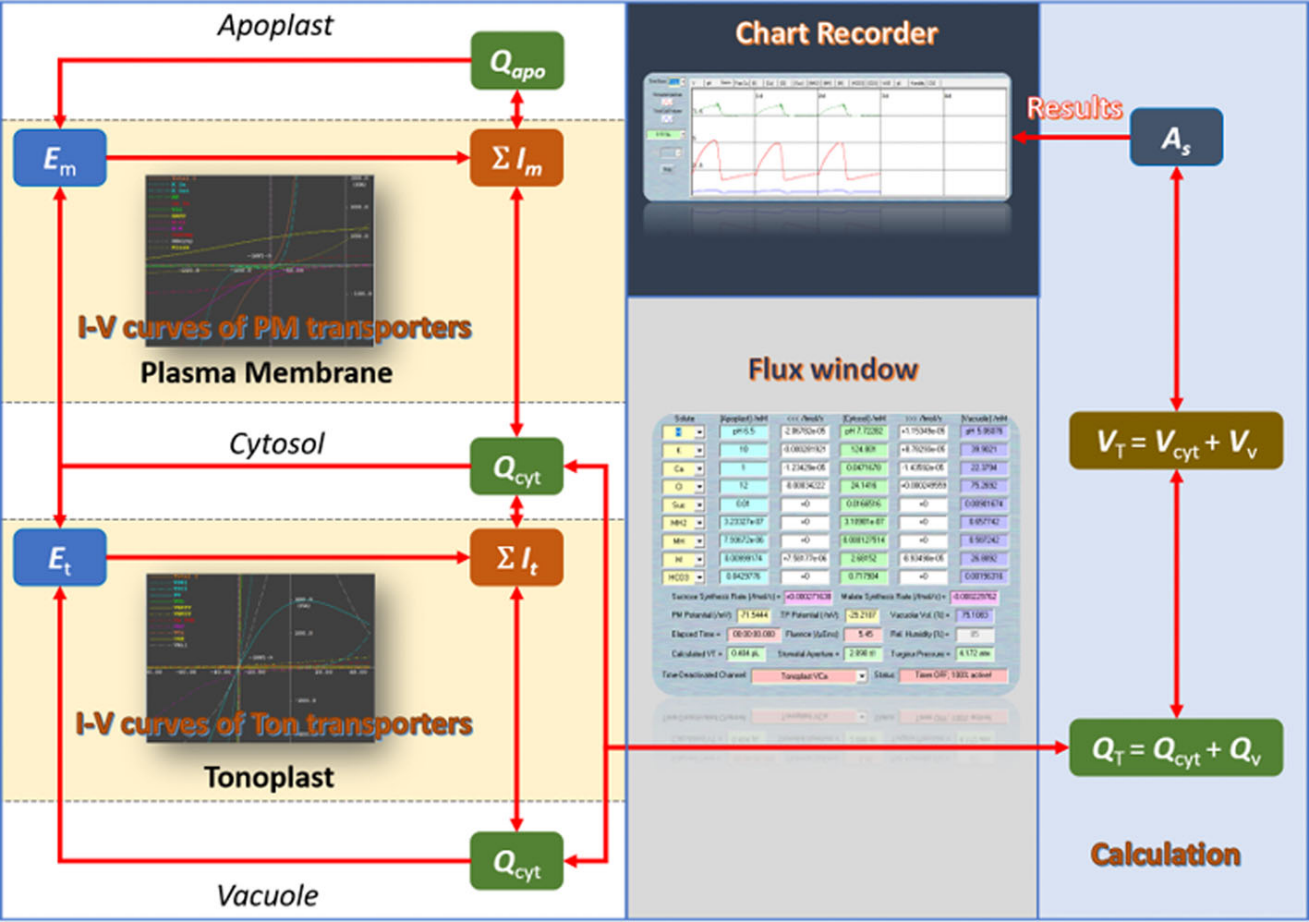
The computational flow of the OnGuard software.

In plants, a complex intracellular signaling network regulates ionic fluxes—mainly K^+^, Cl^−^, and malate—across both plasma membrane and tonoplast, which adjusts the osmotic load and turgor pressure of guard cell that drive the opening and closing of stomata (Blatt, 2000; Hetherington, 2001; Schroeder et al., 2001). Our deep knowledge of this network has made guard cells one of the best-known models in plant cell biology for studying membrane transport, signaling, and ion homeostasis (Willmer and Fricker, 1995; Zhang et al., 2014; Murata et al., 2015; Eisenach and De Angeli, 2017; Jezek and Blatt, 2017; Wang et al., 2018; Sussmilch et al., 2019). However, predicting the full breadth of stomatal behavior from this body of knowledge is much more difficult than had been expected (Blatt et al., 2014). To resolve this, quantitative mathematical modeling offers an effective *in silico* approach to test whether information obtained at the molecular and cellular levels could explain and predict stomatal function. Only recently has an integrated mathematical model of guard cell and stomatal dynamics been developed (Chen et al., 2012; Hills et al., 2012; Wang et al., 2012), and encoded in the OnGuard software platform. In brief, OnGuard relies on a set of interrelated class structures. The Compartment Class has three sub-classes representing the apoplast, the cytosol, and the vacuole, respectively. Each of the sub-class includes data representing its volume (*V*), osmotic solute concentrations, and buffering system for pH and cytosolic Ca^2+^. Then, these data are used to determine the overall guard cell volume, turgor pressure (*Q*), and stomatal aperture (*A*). The Membrane Class represents the plasma membrane and tonoplast, respectively. Each membrane class includes a set of data representing its voltage and a set of functions to calculate this voltage (*E*) from the ionic currents (*I*). The Transporter Class is the core part of OnGuard. It includes the an array of solute transporters, such as ion channels, pumps, exchangers, and so on, in the membranes, as well as relevant functions to calculate the transmembrane fluxes for each transporters. The main advantage of this structured model is that it allows the necessary flexibility and ease to add/remove objects such as selected transporters, and to carry out numerical calculations in a way that enables each cell component to interact within the network of pathways in the model. **Figure 1** demonstrates the computational flow of OnGuard model and, **Table 1** summarizes the details of all transporters in the OnGuard2 (for details please see Hills et al.,2012). OnGuard incorporates all of the fundamental properties in guard cell membrane transport and metabolism, and is sufficient to accurately simulate a wide range of physiological activities of guard cells and corresponding stomatal movements. It has been shown capable of the real predictive power needed to yielded many unexpected outputs (Chen et al., 2012; Wang et al., 2012) and testable results (Chen et al., 2012; Wang et al., 2012; Wang et al., 2014a; Minguet-Parramona et al., 2016; Wang et al., 2017; Jezek et al., 2019). The advances made with OnGuard have led to a more profound understanding of the complex regulatory mechanisms underpinning how guard cells respond to the environmental changes (Wang et al., 2017).

**Table 1:** Predominant plasma membrane and tonoplast transporters used for the OnGuard modeling.

PLASMA MEMBRANE						
**TRANS** **PORTER**	GENE*	**FUNCTION**	**VOLTAGE GATE**	**LIGAND GATE**	**LIGHT GATE**	
**PM K_in_ channel**	KAT1, KAT2	K^+^ uptake	δ=+1.8; V_1/2_=−180 mV	(Ca^2+^)_i_; Cytosolic H^+^; Apoplast H^+^	—	
**PM K_out_ channel**	GORK	K^+^ release	δ=+2	Cytosolic H^+^	—	
**R-Type Anion Channel**	ALMT12, QUAC1	Cl^−^ and Mal release	δ=−2	(Ca^2+^)_i_; Cytosolic H^+^	—	
**Anion VIC**	SLAC1	Cl^−^ and Mal release	—	(Ca^2+^)_i_; Cytosolic H^+^	—	
**V-Gated Ca-IN**	—	Ca^2+^ entry	δ=+1	(Ca^2+^)_i_	—	
**4-State Slayman Hx1**	AHA1, AHA2, *etc*	H^+^ extrusion	—	(Ca^2+^)_i_	Y	
**H:Cl Symport**	NRT1.1, NRT2.1	Cl^−^ uptake	—	—	—	
**H:K Symport**	KUP, HAK, *etc*	K^+^ uptake	—	—	—	
**HMal symp**	ABCB14	Malate uptake	—	—	—	
**Slaymanesque Ca pump**	ACA8, ACA10, *etc*	Ca^2+^ extrusion	—	(Ca^2+^)_i_	Y	
**TONOPLAST**						
**TPK1**	TPK1, KCO3	K^+^ exchange	—	(Ca^2+^)_i_; Cytosolic H^+^	—	
**FV K Channel**	—	K^+^ exchange	δ=+1; V_1/2_=−30 mV	(Ca^2+^)_i_; Cytosolic H^+^	—	
**TPC1**	TPC1	Ca^2+^ and K^+^ influx	δ=+2	(Ca^2+^)_i_; Vacuolar H^+^	—	
**VCL**	ALMT9	Cl- and mal exchange	δ=−1	(Ca^2+^)_i_	—	
**ALMT-Mal**	ALMT6	Mal exchange	δ=−2	(Ca^2+^)_i_; Cytosolic H^+^	—	
**Tonoplast VCa**	-	Ca^2+^ release	δ=+4	(Ca^2+^)_i_	—	
**Vacuole H-ATPase**	VHA-A, VHA-B, *etc*	H^+^ uptake	—	—	Y	
**Vacuole H-PPase**	AVP1, AVP2	H^+^ uptake	—	(Ca^2+^)_i_; Cytosolic K^+^	Y	
**Vac. CLC**	CLC-A, CLC-B, *etc*	Anion exchange	—	Cytosolic H^+^	—	
**Slaymanesque Ca pump**	ACA4, ACA11	Ca^2+^ uptake	—	(Ca^2+^)_i_	Y	
**CAX**	CAX1, CAX2, *etc*	Ca^2+^ exchange	—	(Ca^2+^)_i_	—	
**NHX**	NHX1, NHX2	Cation exchange	—	—	—	

*Genetic codes related to Arabidopsis

Since its official release in 2012, OnGuard has undergone two major updates. In 2017, theOnGuard2 was launched that incorporated with evapotranspiration and the water relations of the whole plant (Wang et al., 2017). It provides a reliable tool to investigate the mechanistic link between guard cell membrane transport and foliar transpiration. Recently, a constraint–relaxation–recovery mechanism (CRR) that simulates the effect of the surrounding epidermal cells on guard cell volume was introduced into the OnGuard2 system (Jezek et al., 2019). As the software upgrades and features updated, several operations is also changed. Here, we describe a standard protocol for using the latest OnGuard2 software in studying stomatal physiology and its related progress of ion transport and homeostasis. To illustrate the functioning of each key options in the software, several examples are given for how to change parameters for ion transporter characteristics as well as humidity. The protocol is simple but encompasses many of the basic concepts associated with stomatal physiology. More importantly, we would like readers to be able to understand the true biological implications of these simulations, and thus, to better use OnGuard platform for their own research in the future. Before starting, we do recommend that readers watch the introductory video online at https://psrg.org.uk/guard-cell-modelling/. We also encourage the beginner to use the current models that we have tested and validated, and to explore and experiment on their own with the model's software to appreciate its use and complexity.

## Materials and Equipment

### Required Hardware

- A computer with 32- or 64-bit processor (Intel i5 or above; or similar AMD productions).
- At least 4 GB RAM and 1 GB of free storage in the hard drive.

We recommend a quad processor system and more memory for best results.

### Required Software

- A Microsoft Windows10 or earlier Windows operating system is required for running the OnGuard software. For the Macintosh system, you must install a parallel system or run the program under Bootcamp with a Windows operating system.
- The OnGuard software (https://psrg.org.uk/guard-cell-modelling/).
- Microsoft Excel or similar software for reading in all the variables in a spreadsheet-readable (*.csv) format
- SigmaPlot, Origin, or other advanced graphic software is required for plotting high-quality graphs of the model outputs and results.

## Methods

### Download and Install the OnGuard Software

1. The OnGuard software can be downloaded at https://psrg.org.uk/guard-cell-modelling/. Choose 32- or 64-bit version (CM-Win32 or CM-Win64) to download, according to your operation system. The installation program's size is around 3 MB.
2. Click the CM-Win64, for example, to call up the HoTsig CM Suite install window. Then click the “Next” button and choose the file for where you want to install the software. Follow the instructions to install the software on your computer. The initialization can be done either automatically or manually. Once finished, you will see the On-Guard icon on your desktop.
  NOTE For first-time users, you will need to register for HoTsig due to the licensing policy of the software. It is free for academic and noncommercial use, but registration required.
3. If you meet any problem with modeling, you could use the “Help” button on the middle top of the main window to find the solutions. Alternatively, you could contact us directly. If you encounter any technical problems with the OnGuard software, please contact on-guard@psrg.org.uk.

### Define the Model

All models are encoded within binary “*.OGB” files. We strongly recommend that you begin using the example models provided with OnGuard, as these models have been extensively tested and validated already. After downloading the software, you will find these files in: This PC\Local Disk (C):\Program Files\HoTsig Cellular Modelling Suite\Examples\ or other folders you manually created when you installed the software.

**Figure 2:**
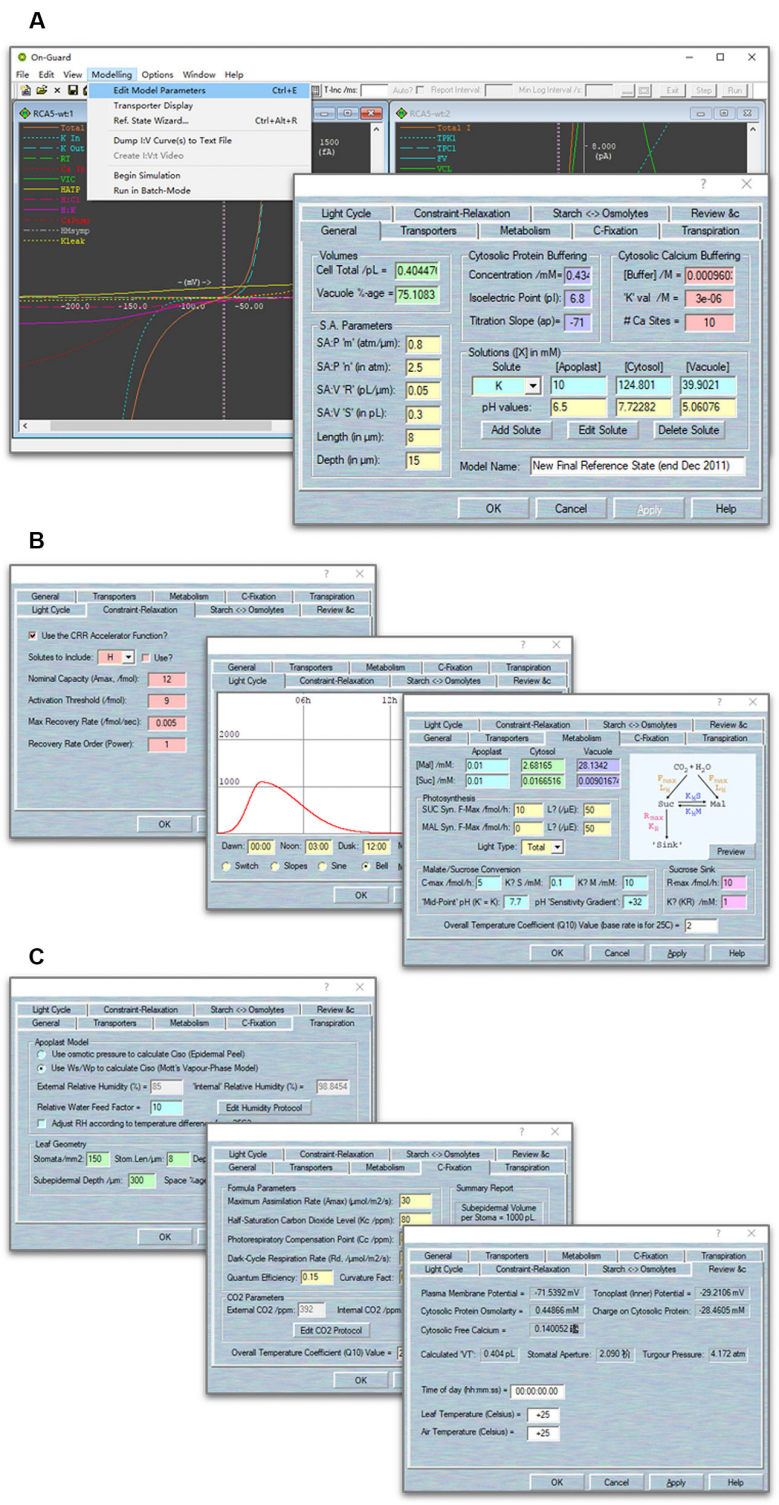
Screenshots of the OnGuard software. **(A)** *Right* The main window with I-V curves' outputs related to membrane transport at the plasma membrane and tonoplast. *Left* The tabular output window detailing the ionic and organic solute contents within each compartment, the fluxes across the plasma membrane and tonoplast, the respective membrane voltages, the macroscopic outputs of cell volume, turgor, and stomatal aperture, and the elapsed time counter. **(B)** Screenshots of selected OnGuard property pages. From right to left: *CRR* function; light cycles; metabolism pages. **(C)** Screenshots of selected OnGuard property pages. From right to left: humidity; C-Fixation; review pages.

You can also define your own custom model. To define the model parameters, use Modelling= > Edit Model Parameters. Several options that can be modified manually (**Figure 2**). To define a model, follow these steps:

1. Populate each membrane with an ensemble of transporters. Use File= > New to open a new model, and use Modelling= > Edit Model Parameters to access the model property pages. The new model opens with a few basic settings (solutes and ion transporters) already in place. You can then add/delete/alter the transporters on the “Transporters” tab, by using the “Delete” and “Modify” buttons to remove a transporter or modify its parameters, respectively. Use the “New Channel”, “New Pump”, “New Carrier”, and “Aquaporins” buttons to create new transporters in each of these categories.
2. Establish the starting solute composition, and set the buffering and macroscopic parameters. Use Modelling= > Edit Model Parameters to access the model property pages, and use the “General” tab to access these parameters. The new model opens with basic settings in place (**Figure 2A**). These values include cell volumes, stomatal aperture (SA) parameters, cytosolic protein and calcium buffering, as well as the concentration of solutes in the apoplast, cytosol, and vacuole. You can change most of these by directly typing into the entry boxes provided.
3. Set the starting metabolic parameters, by using Modelling= > Edit Model Parameters to access the model property pages, clicking on the “Metabolism” tab to access these parameters (**Figure 2B**). The new model opens with the default settings in place. Note that you can access all of the photosynthesis, malate/sucrose conversion and sucrose catabolism parameters from the Preview page and, at the same time, visualize the rough characteristics for synthesis/catabolism over a 24-hour period.
4. Choose a light cycle. Use Modelling= > Edit Model Parameters to access the model property pages, and use the “Light Cycle” tab to access these parameters. The model opens with default settings in place for a 24-hour cycle (**Figure 2B**). Use the Minimum Intensity and Maximum Intensity together with the Dawn, Noon, and Dusk settings to adjust the light cycle or to set it to a continuous, fixed value. You could also select different sources of light under which your experiments would be run (see the example below).
5. Chose the humidity cycle. Use Modelling= > Edit Model Parameters to access the model property pages, and use the “Transpiration” tab to access these parameters. The new model opens with basic settings in place (**Figure 2C**). Choose “Use W_s_/W_p_ to calculate C_iso_ (Mott's Vapour-Phase Model)”. You could adjust the *RWF* (relative water feed) setting to manipulate the sensitivity of stomata to environmental relative humidity (RH), and simulate stomatal changes under water stress. Use the “Edit Humidity Protocol” to set the humidity level. “Leaf Geometry” provides settings to define the leaf geometry of your study plants.
6. Choose the CO_2_ cycle. Use Modelling= > Edit Model Parameters to access the model property pages, and use the “C-Fixation” tab to access these parameters. The new model opens with basic settings in place. Use the “Edit CO2 Protocol” to adjust the CO_2_ as you had setting the humidity cycle.
7. Choose the constraint-relaxation. Use Modelling= > Edit Model Parameters to access the model property pages, and use the “Constraint-relaxation” tab to access these parameters (**Figure 2B**). The new model opens with basic settings in place. Use the “CRR Accelerator Function” to accelerate the rate of stomatal opening.
8. Set the starting time. Use Modelling= > Edit Model Parameters to access the model property pages, and use the “Review” tab to set the time of day and to review the model parameters (**Figure 2C**).

NOTE For first-time users, we strongly recommend using the default settings in the software model. Once you are familiar with running the software and understand its operations, you can easily explore the influence of changing any values according to your own research objective.

### Establishing a Reference State

An iterative commutating model, like OnGuard, does not define final end-points. Therefore, an initial point is required for calculating the time increment and dynamics of the model. The best initial point of guard cell model is the “closed state”, that is in the dark, when no net changes occur in solute fluxes within the cell. Thus, OnGuard incorporates a Reference State Wizard to help determine such an initial point. The Reference State Wizard allows you to review and edit all the parameters in the system, including solute compositions and membrane potentials, and to establish a stable, homeostatic steady state for the model. It provides several pages for comparing the solutes fluxes across the plasma membrane and tonoplast, and balances the ionic species by adjusting associated transporters populations and kinetic characteristics.

**Figure 3:**
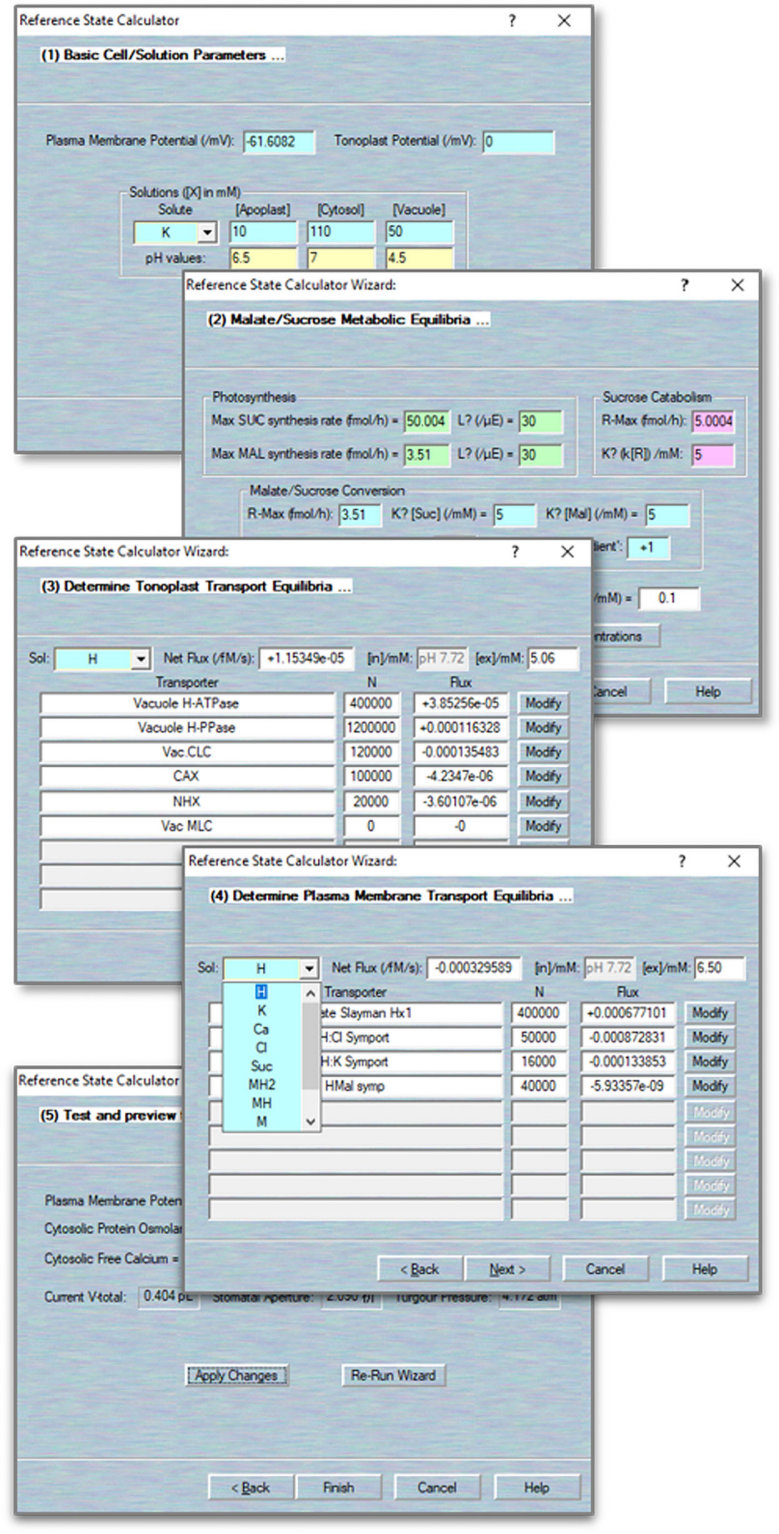
Define/modify transporters. Sample pages for accessing the biophysical and kinetic parameters foreach of the various transporters, including the ion channels, pumps and carriers, and aquaporins.

1. Activate the wizard tool. Use Modelling= > Reference State Wizard to activate this wizard. The wizard comprises a run-through series of pages, each of which permitting modifications to different sets of parameters. Use the “Back” and “Next” buttons to move among the pages. The first page gives access to the solute composition in each compartment as well as the membrane voltage. Membrane voltage is accessible only on this page, whereas solute composition can be adjusted again in later pages (**Figure 3**).
2. Adjust the metabolism. Click “Next” to access this page. The parameters here are identical to those accessed through the Edit Model Parameters= > Metabolism tab (**Figure 3**).
3. Adjust the tonoplast transport balance. Click “Next” to access the page. Here, selecting a solute from the drop-down list (top left) calls up all known existing transporters that can carry the solute (**Figure 3**).
  NOTE We recommend beginning with non-driver ions (K^+^, Cl^−^, Ca^2+^, Mal^2−^, sucrose) and only then returning to adjust the balance of H^+^ with the H^+^-ATPase and H^+^-PPase. For each transporter, you have access to the number of transporters, N, and a read-out of the corresponding flux in units of fmol/s. Total flux is indicated in the box beside the solute selection box along with the prevailing solute concentrations (top right). In most cases, you can adjust values of N for each transporter as well as the compartment solute composition. Click on any other active box to update the changes made on this page. Click on the “Modify” buttons beside each transporter to access their respective parameters.
**NOTE** For each ion, there MUST be at least one pathway for influx and one pathway for efflux. For example, if you set up a K^+^ uptake pathway (e.g., the KAT1 channel) on the plasma membrane, you must add a way for K^+^ to exit (e.g., *via* the GORK channel) to balance the ionic species and charges across the membrane.
**NOTE** We find it sufficient to bring the net flux of each solute to within ±10^−6^ fmol/s through adjustment of transporter numbers (N) and solute composition. In this way, the simulations will reach a stable reference state within the simulation time of 8–10 h.
4. Adjust the plasma membrane transport balance. Click “Next” to access this page and follow the same procedure as in 1–4 (**Figure 3**).
5. Apply those changes. Click “Next” and review the macroscopic parameters. You must click the “Apply Changes” button to complete the process. You may find it necessary to repeat the wizard cycle two or three times before the system settles upon an acceptable starting point. Use the “Re-Run Wizard” button to repeat the cycle. Use the “Finish” button to exit the wizard (**Figure 3**).

Now you are ready to run the model you set up (see below for how to run the model). Once you are happy with this setting, select the “Review &c” button and adjust the “Time of day” to zero. This allows you to reset the start time in the simulation. **NOTE** We strongly recommend to users that they rename the new model, by clicking the “File” button and use “Save as” tab to avoid overwriting the original model.

### Running the Model

**Figure 4:**
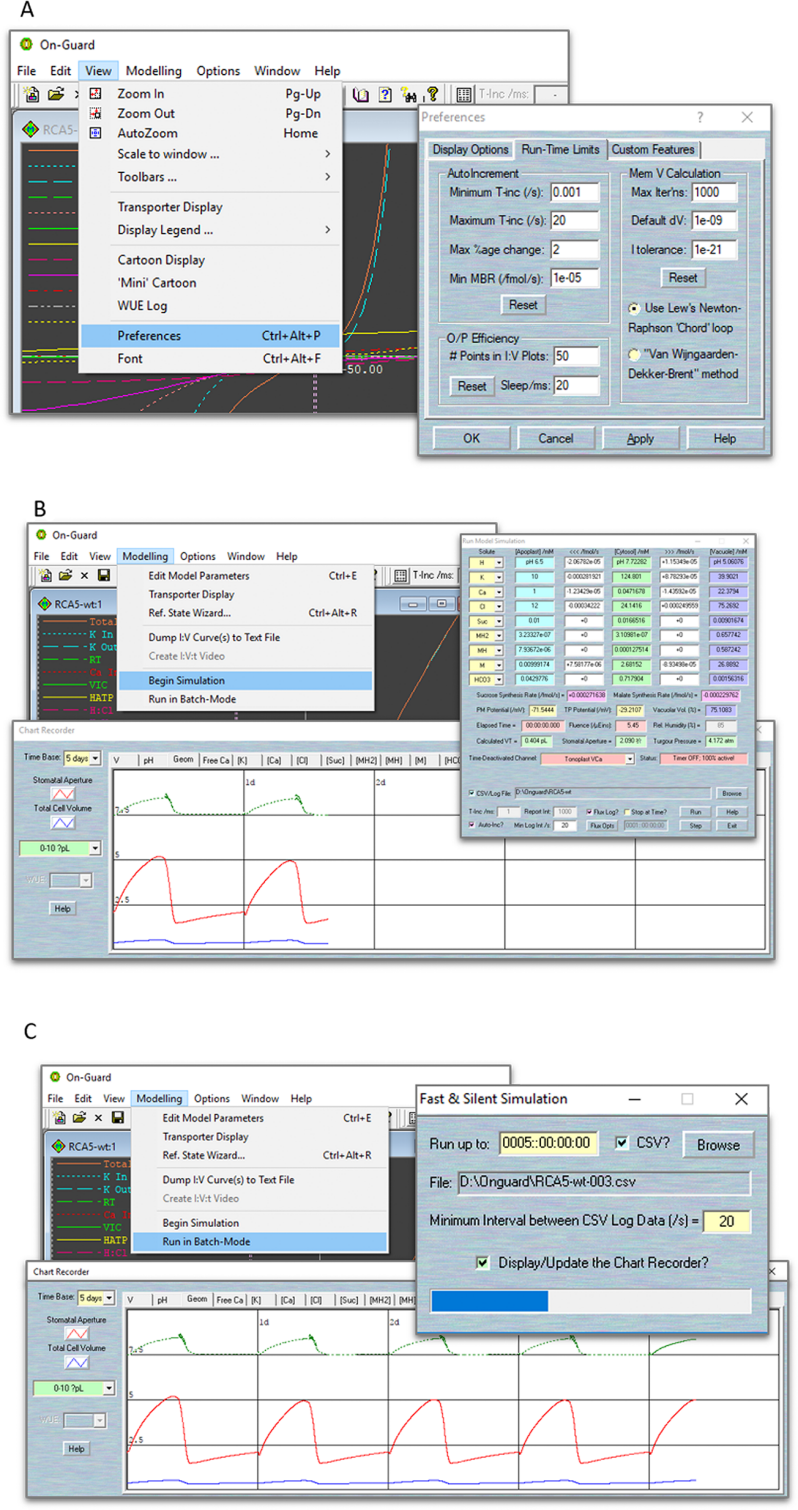
Screenshots of the OnGuard settings. **(A)** Editing the parameters that determine the variable time-interval limits, the range of permissible variable changes per time interval, and the tolerances for the free-running voltage and current estimates. **(B)** The normal running mode of OnGuard. **(C)** The fast running mode of OnGuard.

When a model simulation is running, your computer's CPU is being asked to do a great deal of work. First, the calculation of the two membrane voltage and the array of solute fluxes involves solving—at the very least—several hundreds of complex equations in each and every cycle of the iterations. In addition, the continuous updating of various display elements (two I-V curves views, all the solute concentration/flux text boxes, and the chart recorder) requires considerable screen output. Last, there is potentially a huge amount of data being written to your computer's disc, in the form of data logging files. If you find your computer “hanging” or crashing during a run, please use the “View” tab and choose “Preferences” in the drop-down list to access “Run-Time Limits” pages. The controls in the “Auto-Increment” tab let you specify the parameters used for calculating the time interval used when running a simulation with the auto-increment option turned on. NOTE We recommend set “Minimum T-inc” and “Maximum T-inc” values to 0.001 and 20 s, respectively, “Max %age change” to 3%; “Max Iterns” to 1000; the “Default dV” to 1 × 10^−9^, and “I Tolerance” to 1 × 10^−21^. In addition, you can speed up your calculations by adjusting the “O/P Efficiency” settings, which enables your computer's processor to achieve the best compromise between speed and quality in producing the graphical displays (**Figure 4A**).

There are two ways to run the model. Use Modelling= > Edit Model Parameters to access the model property pages,

1. Use the “Begin Simulation” tab to run your model. With this option, the tabular flux output window and graphical chart-recorder output window will appear (**Figure 4B**). The flux output window gives detailed information of each solute's content in the cytosol and vacuole, its fluxes across the two membranes, their membrane potentials, the macroscopic characters of cell volume, turgor pressure and stomatal aperture, as well as the consumption time counter.
**NOTE** We highly recommend that users activate the “Auto-increment” tick-box in the flux output window, and therein set the “Min Log Interval” to 20 s. This allows your computer to obtain an appropriate temporal resolution in the data-logging phase.
  Then use the “Run” button to start running the model. You will see the timely model outputs on the chart-recorder window. This window provides a tabular selection of cytosolic and vacuolar solute components, pH and (Ca^2+^), and each membrane's potentials and so on. Tick the “CSV/Log file” to create the file in which to store your results. These data will log into *.csv file that is readable by almost all spreadsheet programs. In addition, you could log the fluxes details from and individual transporter by clicking the “Flux Log?” tick-box and choosing the outputs that interest you from the “Flux Opts” tab. You may also tick “Stop at Time?” to choose the when you want the model stopped (**Figure 4B**).
**NOTE** OnGuard normally records the content of each solute in different compartments, as well as the net flux across the plasma membrane and tonoplast. It will also record the fluxes from each transporter, cytosolic and vacuolar (Ca^2+^) and pH level, membrane potentials, metabolic synthesis rates, and macroscopic variables such as stomatal conductance and transpiration. Only a small portion of these variables is displayed on the chart recorder screens during the actual simulations. You can access more details from the saved *.csv files, which could then be read by spreadsheet programs (such as the Microsoft Excel or others). For a more comprehensive visualization of your datasets, we suggest using high quality graphing software, such as SigmaPlot.
2. Use the “Run in Batch-Mode” tab to run your model quickly without any full screen updating. With this option, you will not see the chart recorder unless you tick “Display/Update the Chart Recorder”. Similar as to “Begin Simulation”, you could choose to log your data into a.csv file, but you may NOT log the flux details when this option is enabled (**Figure 4C**).

NOTE We have found it most useful to introduce changes to a model after running simulations for 3 days in model time. This approach has the advantage of providing a baseline of logged data in the control situation (or Reference State) within the same data set as the condition(s) to be tested.

## Simulation Results and Discussion

The functions and characteristics of ion transporters play key role in guard cell regulation. Guard cells open stomatal pores through the transport and accumulation of osmotic solutes to promote water uptake and cell volume expansion. They close these pores by releasing such solutes through ion transporters at the plasma membrane. Any changes to these transporters may greatly affect stomatal movements. For example, eliminating the plasma membrane Cl^−^ channel, SLAC1, considerably slows the stomatal closure and opening, also affecting the channel activities of both inward- and outward-rectifying K^+^ channels (Wang et al., 2012). The two dominant mutations of H^+^-ATPase (*ost2*) result in a constitutive activity of H^+^-ATPase and insensitive to both ABA and Ca^2+^, leaving these plants unable to close their stomata (Merlot et al., 2007). In the following, we use several classic cases to demonstrate how OnGuard2 system operates. It would be grateful that our readers could understand the true biological implications behind these simulations.

### Simulation of Mutations *via* Changes to the Transporter Populations

*In silico* simulation of the steady-state levels of transporter expression, such as over- and reduced-expression lines, the knock-out mutations, *etc*., can be easily achieved in OnGuard2 system through manipulating transporter population settings. Here two examples will be used to demonstrate how to simulating the knock-out and overexpression mutations.

**Figure 5:**
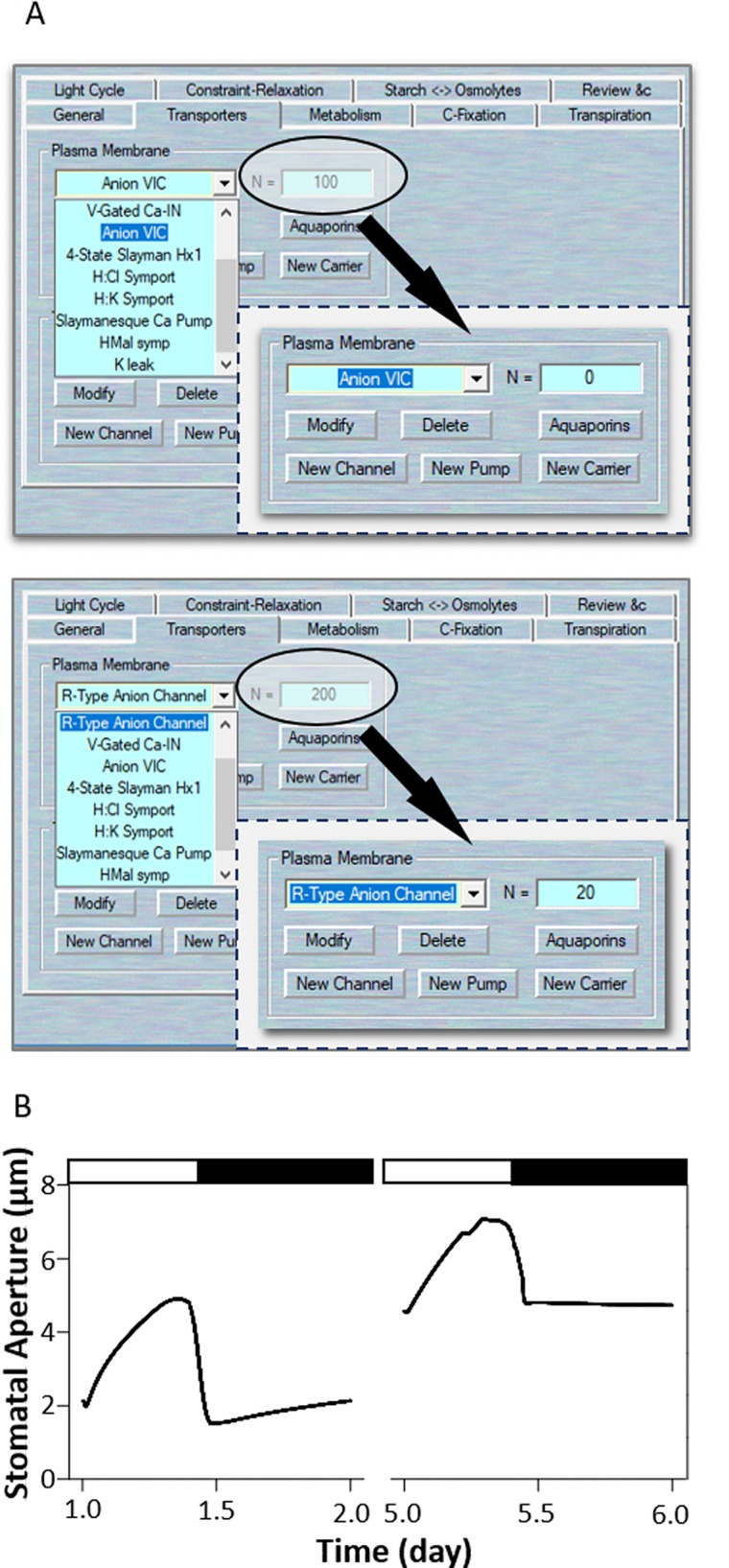
Modeling the *slac1* mutant. **(A)** Manipulation of ion channel characteristics. **(B)** Stomatal aperture outcomes modeled with OnGuard software. Outcomes resolved over a diurnal cycle (12-hour light:12-hour dark) with 10 mM of KCl, 1 mM of CaCl_2_, and pH 6.5 outside the guard cell (Chen et al., 2012). Representative diurnal cycles are shown for (*left*) the wild type and (*right*) the *slac1* mutant.

In 2012, Wang et al. (2012) successfully employed original OnGuard system to investigate the counterintuitive alterations in K^+^ channel activity in the *slac1* anion channel mutation. The SLAC1 channel is a main pathway for the release of anions from guard cells during stomatal closure in plants (Negi et al., 2008; Vahisalu et al., 2008). To modeling this mutant with OnGuard2, first load the example RS-Arbidopsis.OGB file. Run the model for three continue diurnal cycles as a control (wt). Use Modelling= > Edit Model Parameters to access the model property pages, and use the “Transporters” tab (**Figure 5A**). Choose the “Anion VIC” channel from the plasma membrane transporters in the drop-down list and change its channel population (N) value from 100 to 0; also, select the “R-type anion channel” and change its' N value to 20 or less (These remaining populations represent the ALMT-like transporter's current in the guard cell). Press “OK” to finish the editing progress. Run the model for another three diurnal cycles, and the *slac1* mutant will be obtained (**Figure 5B**). The full results will be logged in *.csv file. (Optional). At the end of this simulation, you could change the N values back to their control values, and run the model again for a further several diurnal cycles to serve as a reference. Eliminating the SLAC1 channel greatly slows stomatal opening and closing rates, stomatal aperture range, and alternating of inward- and outward-rectifying K^+^ channel activities (Wang et al., 2012). **Figure 5B** shows that OnGuard2 also faithfully reproduced the stomatal characteristics of the wild-type plant and *slac1* mutant. The *slac1* model had a substantial effect upon the stomatal dynamic range over the diurnal cycles (**Figure 5B**), the form of large increases in cytosolic and vacuolar osmotic solutes, including K^+^, Cl^−^, and malate (Figure 4 and Figures S2 and S7 in Wang et al., 2012). These effects are due to the excessive accumulation of anions in the mutant, thereby affecting the cytosolic level of H^+^ and (Ca^2+^), both of which are known to regulate the activity of inward- and outward- rectifying K^+^ channels (see the analysis in Wang et al., 2012).

**Figure 6:**
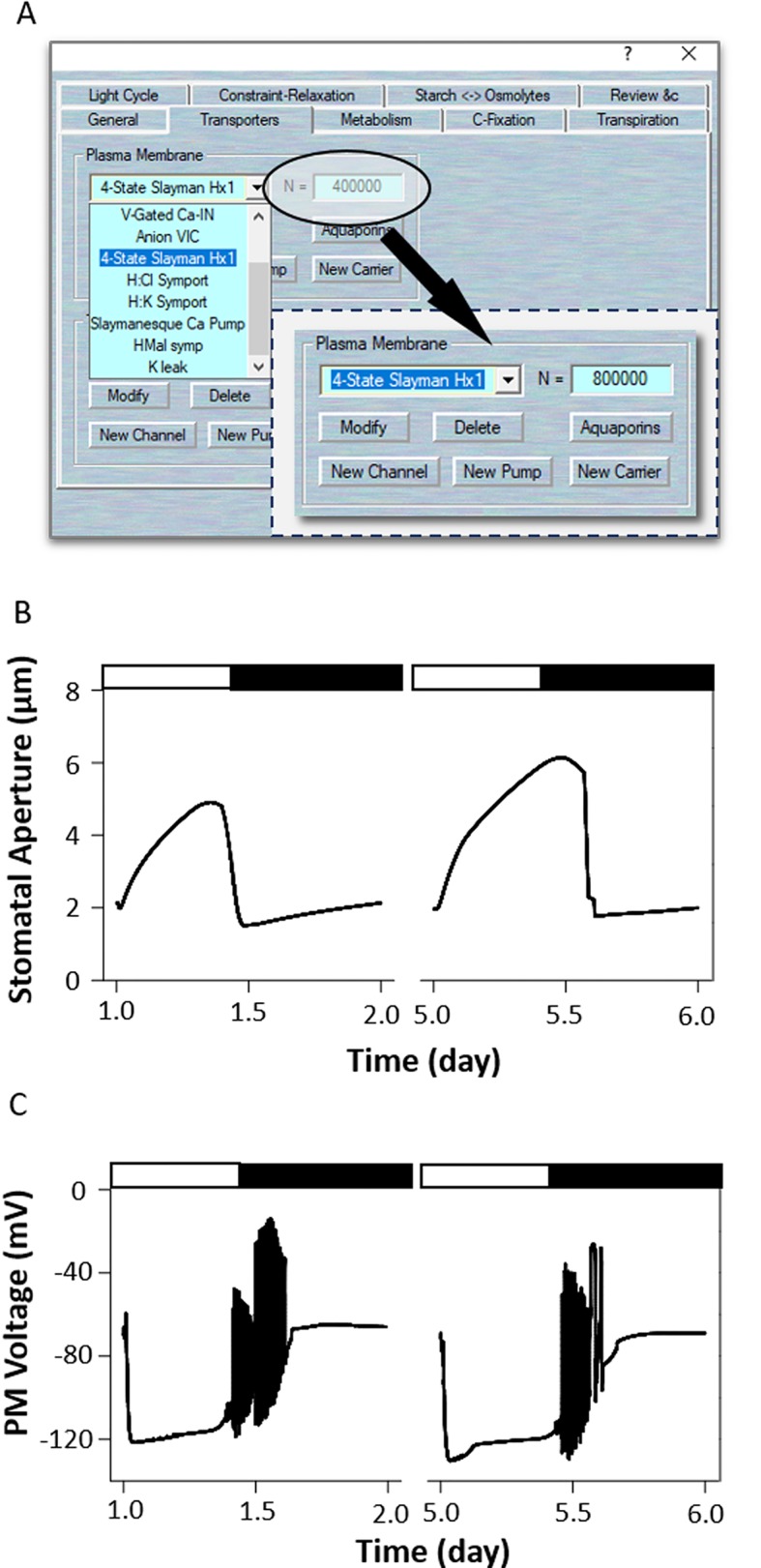
Modeling the *oxHATPase* mutant. **(A)** Manipulation of ion transporter characteristics. **(B)** Stomatal aperture and **(C)** plasma membrane voltage outcomes modelled with OnGuard software. Outcomes resolved over a diurnal cycle (12-hour light:12-hour dark) with 10 mM of KCl, 1 mM of CaCl_2_, and pH 6.5 outside the guard cell (Chen et al., 2012). Representative diurnal cycles are shown for (*left*) the wild type and (*right*) the *oxHATPase* mutant.

In light, H^+^-ATPase was activated to hyperpolarization of the plasma membrane, allowing K^+^ uptake through inward-rectifying K^+^ channels that open stomata (Jezek and Blatt, 2017; Wang et al., 2018). Overexpression of H^+^-ATPase had a significant effect on light induced stomatal opening and enhance plant growth in *Arabidopsis* (Wang et al., 2014b). To modeling this mutant, first load the example RS-Arbidopsis.OGB file. Run the model for three continue diurnal cycles as a control (wt). Use Modelling= > Edit Model Parameters to access the model property pages, and use the “Transporters” tab (**Figure 6A**). Choose the “4-State Slayman Hx1” channel from the plasma membrane transporters in the drop-down list and change its channel population (N) value from 400,000 to 800,000. Press “OK” to finish the editing progress. Run the model for another three diurnal cycles, and the *oxHATPase* mutant will be obtained (**Figure 6B**). The full results will be logged in *.csv file. (Optional). At the end of this simulation, you could change the N values back to their control values, and run the model again for a further several diurnal cycles to serve as a reference. Wang et al. (2014b) reported that overexpression of the H^+^-ATPase (AHA2) driven by a guard cell-specific promoter significantly enhanced stomatal conductance and opening in the light. OnGuard2 faithfully reproduced stomatal behavior in the overexpression plants (**Figure 6B**). Increased the expression of H^+^-ATPase elevates cytosolic pH, enhances H^+^ flux through coupled transport and malate metabolism. It also promotes light induced hyperpolarization of the plasma membrane (**Figure 6C**), which enhances the activities of voltage-gated inward-rectifying K^+^ channels effectively induce K^+^ uptake into guard cells and stomatal opening.

### Simulation of Mutations *via* Changed Channel Characteristics

OnGuard2 has a set array of characteristics for each transporters. For example, the fundamental gating characteristics for the plasma membrane K^+^ channels include overlapping values for gating charge (δ) and half-activation voltage (V_1/2_), as well as the sensitivities to extracellular (K^+^), cytosolic pH, and (Ca^2+^)_i_ (Hills et al., 2012). Manipulation these parameters has substantial effect on channel properties and the ion fluxes through this transporter. In the following, we take the *ost2* example to demonstrate how to manipulate transporter characteristics on OnGuard2.

**Figure 7:**
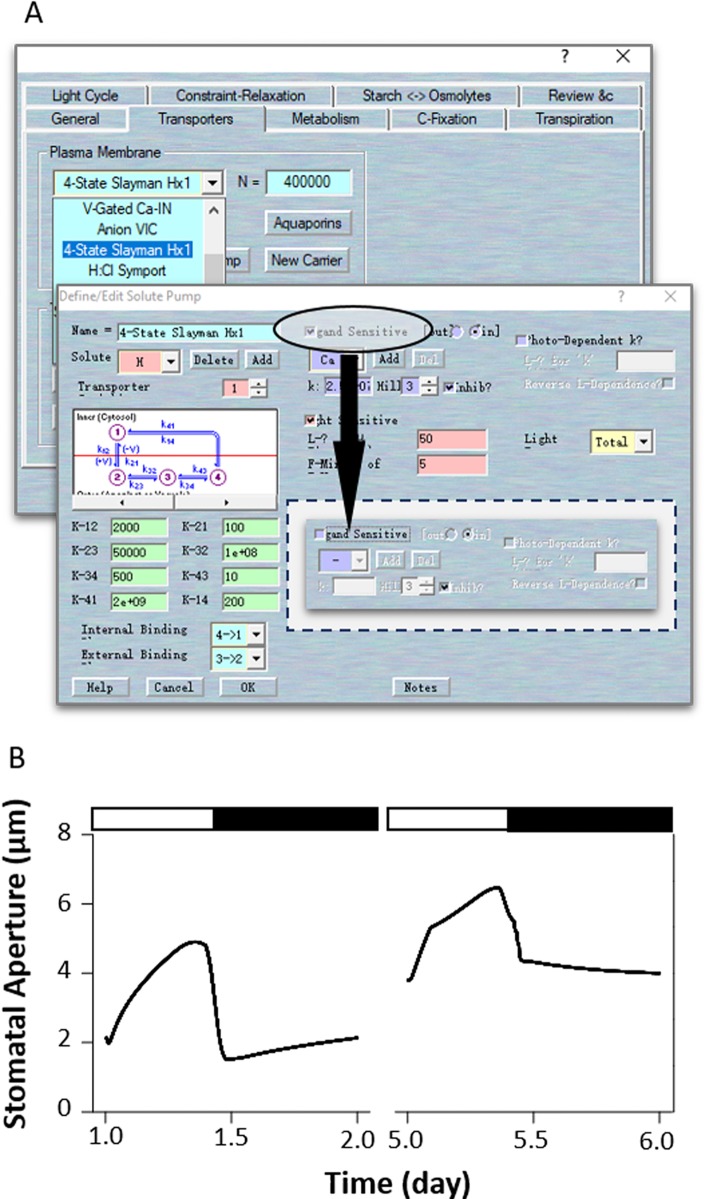
Modeling the *ost2* mutant through uncoupling (Ca^2+^)_i_-sensitivity. **(A)** Manipulation of pump characteristics. **(B)** Stomatal aperture outcomes from the OnGuard software. Outcomes resolved over a diurnal cycle (12-hour light:12-hour dark) with 10 mM of KCl, 1 mM of CaCl_2_, and pH 6.5 outside the guard cell (Chen et al., 2012). Representative diurnal cycles are shown for (*left*) the wild type and (*right*) the *ost2* mutant.

Merlot et al. (2007) identified two dominant mutants at the OST2 (OPEN STOMATA 2) locus that give to rise phenotypes with enhanced and constitutive activity of the H^+^ pump and insensitivity to ABA. Interestingly, the *ost2* mutants exhibit larger stomatal apertures and are insensitive to an elevated external Ca^2+^ concentration (Merlot et al., 2007). Elevated external (Ca^2+^) exposure is known to increase cytosolic (Ca^2+^), so the insensitivity to (Ca^2+^) changes suggests a loss in the Ca^2+^-dependence of the H^+^-ATPase (Kinoshita et al., 1995) may be an important consequence of these *ost2* mutations. Based on this, we simulated *ost2* through an uncoupling of H^+^-ATPase sensitivity to (Ca^2+^)_i_ (**Figure 7A**). To do this, first load the example RS-Arbidopsis.OGB file. Run the model for three continue diurnal cycles as the control (wt). Use Modelling= > Edit Model Parameters to access the model property pages, and click on the “Transporters” tab (**Figure 7A**). Choose the “4-State Slayman Hx1” transporter in the plasma membrane drop-down list and click “Modify” to enter the transporter edit window. Click on the tick-box to deselect the “Ligand-sensitive” for Ca^2+^ (**Figure 7A**). This setting makes the H^+^-ATPase independent of any (Ca^2+^)_i_ changes. Press “OK” to finish the editing progress. Then Run the model for another three diurnal cycles to obtain the *ost2* mutant (**Figure 7B**). The full results will be logged into a *.csv file. Uncoupling the (Ca^2+^)_i_ sensitivity of the H^+^-ATPase in the model significantly increased stomatal aperture in the dark, slowed the stomatal opening and closing rate, reduced the dynamic range of the apertures (**Figure 7B**), and hyperpolarized the membrane voltage (see **Figure 2** of Blatt et al., 2014), much as Merlot et al. (2007) reported.

**Figure 8:**
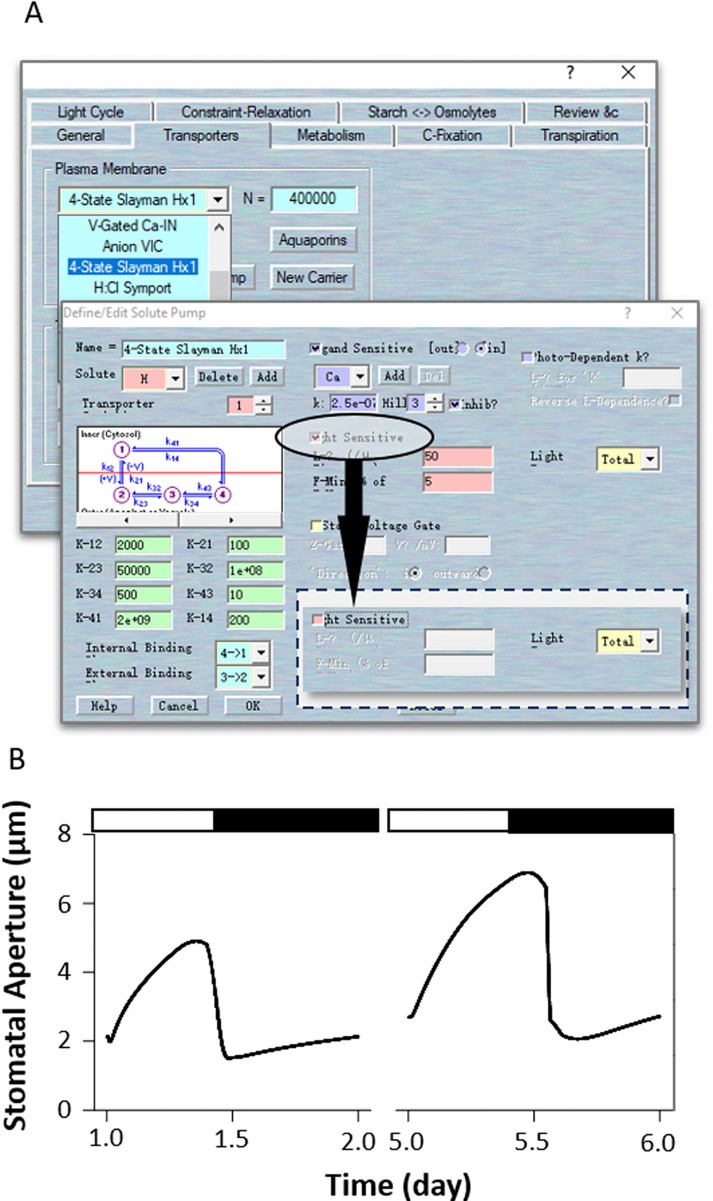
Modeling the *ost2* mutant through uncoupling light-sensitivity. **(A)** Manipulation of pump characteristics. **(B)** Stomatal aperture outcomes from the OnGuard software. Outcomes resolved over a diurnal cycle (12-hour light:12-hour dark) with 10 mM of KCl, 1 mM of CaCl_2_, and pH 6.5 outside the guard cell (Chen et al., 2012). Representative diurnal cycles are shown for (*left*) the wild type and (*right*) the *ost2* mutant.

According to experimental observations, there are other ways to simulate the effects of *ost2* on H^+^-ATPase activity. For example, *ost2* mutant showed insensitivity to darkness, suggests it might have postulated that the H^+^-ATPase is uncoupled from regulation by light, and hence from ATP turnover (Chen et al., 2012). In this case, use Modelling= > Edit Model Parameters to access the model property pages, and use the “Transporters” tab. Chose the “4-State Slayman Hx1” transporter in the plasma membrane drop-down list and click “Modify” to enter transporter edit window. Click on the tick box on the middle right to deselect the “Light sensitive” parameters for (**Figure 8A**). In this simulation, uncoupled the light sensitivity of the H^+^-ATPase led to a huge enhanced stomatal aperture in the daytime, followed with a deep decreased with light transit. However, the stomatal closure is not affected (**Figure 8B**). Obviously, modeling the *ost2* mutations through manipulation of the light-sensitivity of the pump in the H^+^-ATPase properties is not sufficient to reproduce the *ost2* mutant behaviors. Therefore, we highly recommend readers to examine your own model according to a comprehensive analysis and comparison of the experimental results and existing data.

### Simulation of Wild-Type Plants’ Response to a Humidity Change

**Figure 9:**
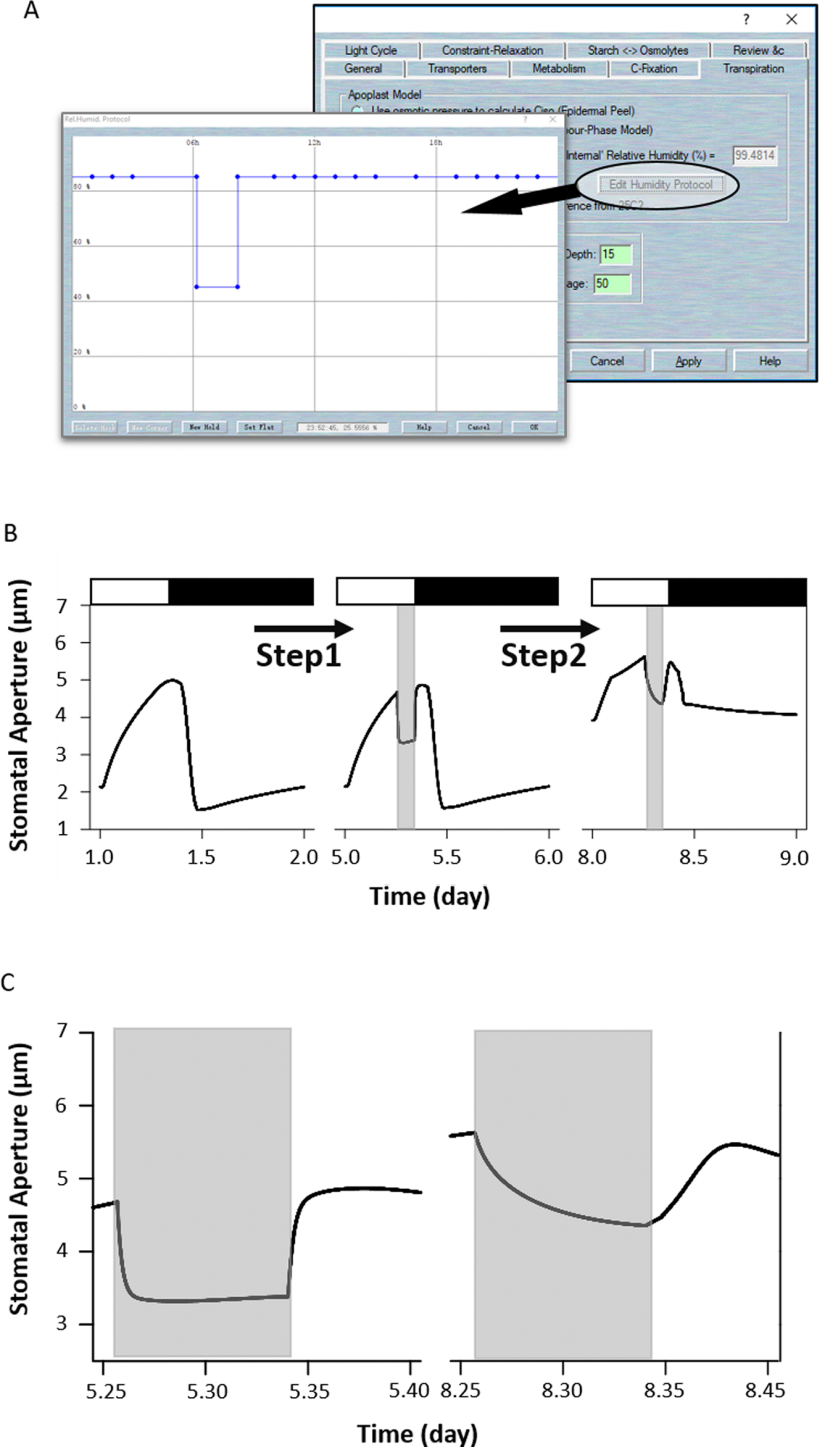
Modeling the wild-type and *ost2* mutant plants in response to humidity. **(A)** Changing the humidity protocol in the model. **(B)** Stomatal aperture outcomes from the OnGuard model simulation. Outcomes resolved over a standard diurnal cycle (12-hour light:12-hour dark) with 10 mM of KCl, 1 mM of CaCl_2_, and pH 6.5 outside the guard cell (Chen et al., 2012). Representative diurnal cycles are shown for (*left*) the wild type and (*middle*) the *ost2* mutant, and (*right*) the *ost2* mutant with high VPD. **(C)** The details of stomatal aperture changes in response to high VPD. *Right*: the wild-type and *left*: the *ost2* mutant.

The response of stomata to air humidity or vapor pressure deficit (VPD) provides another modeling example. Widening the VPD leads to a proportional increase in the stomatal transpiration rate of plants. Therefore, stomata will close their pores under high VPD conditions to avoid excessive losses of water, especially when the plant already has less access to water in the soil. To simulate response to humidity in the OnGuard system, Wang et al. (2017) integrated a new set of variables and parameters related to leaf water relations that linked whole-plant transpiration to the guard cells' molecular functions. To simulate this humidity response, first load the example RS-Arbidopsis.OGB file. Run the model for three continue diurnal cycles. Then sse Modelling= > Edit Model Parameters to access the model property pages, and click on “Transpiration” tab to access the plant water relations. Select the button for “Use Ws/Wp to calculate Ciso” (see **Figure 1C**). NOTE *RWF* indicates the relative amount of water fed to the leaf. When *RWF* is set to 10 or less the simulation is equivalent to a water-stressed plant; when *RWF* is 40 or greater, it is equivalent to a well-watered plant. Click the “Edit Humidity Protocol” to call up the protocol page. Create a humidity step going from 85% to 40% RH for a period of 2 h during the daylight period, and use “OK” to finish the editing progress (**Figure 9A**). Run the model for another three diurnal cycles (**Figure 9B**). The full results will be logged into a *.csv file.

OnGuard2 faithfully reproduced stomatal behavior in plants responding to VPD. Increasing the VPD in the model led to rapid stomatal closure in wild-type plants (**Figure 9B**). In our simulation, the decrease in stomatal aperture was accompanied by an increase of osmotic solutes caused by the water lost from, and reduced volume of guard cell (see **Figure 3** of Wang et al., 2017). Changes in volume are tightly associated with ion transport by affecting both cytosolic and vacuolar ion content, particularly through changes in (Ca^2+^)_i_ and pH_i_, which are known to affect osmotic fluxes across the plasma membrane (Alleva et al., 2006; Verdoucq et al., 2008; Bellati et al., 2010; Maurel et al., 2015). OnGuard also predicted a marked changes in (Ca^2+^)_i_ and pH_i_ associated with stomatal movements in response to high VPD (Wang et al., 2017). The elevated (Ca^2+^)_i_ affects on all fluxes mediated by the transporters that are sensitive to (Ca^2+^)_i_, and, consequently, upon the total osmotic solute contents within the guard cells. It also influences these water fluxes by regulating the water channels (Wang et al., 2017). Finally, as (Ca^2+^)_i_ reduces the water flux rate, it also affects stomatal conductance, transpiration, and water vapor pressure. Accordingly, OnGuard2 attributes the increase in pH_i_ to altered concentration of malate (Wang et al., 2012; Jezek and Blatt, 2017).

### Simulation of the *ost2* Mutant Response to Changed Humidity

The steps: First load the example RS-Arbidopsis.OGB file. Run the model for three successive diurnal cycles. Use Modelling- > Edit Model Parameters to access the model property pages, and use the “Transpiration” tab to access the plant water relations. Select the button for “Use Ws/Wp to calculate Ciso”. Click on the “Edit Humidity Protocol” to call up the editable window for creating a protocol that has a step to 40%RH for a period of 2 h (**Figure 9A**), and Use “OK” to finish the editing progress. Run the model for another three diurnal cycles. As the result above, the stomata closed quickly in response to the humidity reduction (**Figure 9C**). Then, use Modelling- > Edit Model Parameters to access the model property pages, and use the “Transporters” tab. Choose the “4-State Slayman Hx1” transporter in the plasma membrane drop-down list and click on “Modify” to enter the transporter edit window (**Figure 7A**). Click on the tick-box in the upper right to deselect the “Ligand-sensitive” parameters for Ca^2+^. Then press “OK” to finish the editing progress. Run the model for another three diurnal cycles.

Uncoupling the H^+^-ATPase sensitivity to (Ca^2+^)_i_ (*ost2* model) enhanced stomatal aperture (**Figure 7B**). It also slowed the kinetics in the aperture with step changes in VPD (**Figures 9B, C**); suppressing both inward- and outward- K^+^ currents (see **Figure 5** of Wang et al., 2017). These effects may be explained by the elevated levels of cytosolic pH and Ca^2+^ (see Figure S3 of Wang et al., 2017). Again, the (Ca^2+^)_i_ elevation can be seen as a result of uncoupling (Ca^2+^)_i_ from the H^+^-ATPase. This leads to a hyperpolarized plasma membrane and increased Ca^2+^ influx (Figure S3 of Wang et al., 2017). Likewise, the rise in pH_i_ arose from uncoupling (Ca^2+^)_i_ of the H^+^-ATPase, thereby facilitating an H^+^ efflux (Figure S3 of Wang et al., 2017). These elevations largely pre-empt the effects on the K^+^ currents of further changes in (Ca^2+^)_i_ and pH_i_ under VPD.

## Conclusion

The guard cell membrane transport mechanism is defined by our current knowledge of a large number of key transporters and their biophysical properties and regulation characteristics. OnGuard software that incorporates these features has already yielded sufficient detailed outcomes to guide phenotypic and mutational studies of plants. It offers users an unprecedented tool to explore both stomatal regulations and behaviors. The advances made in homeostatic models such as OnGuard lies in their ability to generalize physiological behavior and to make experimentally verifiable predictions. To make the progress of simulation progression and prediction generation as simple and intuitive as possible, the OnGuard software enables users to easily access their settings and parameters during the modeling process, allowing users to restructure the model by editing its model elements according to one's own research work and project design. In this report, we used several classic examples to introduce a standard protocol to simulate stomatal behavior in wild-type and mutated plants, as well as their responses to changed humidity levels. We encourage users to adopt OnGuard software for their own applications when seeking to relate guard cell membrane transport, homeostasis, and stomatal behavior and to give feedback to help us improve our presented model.

## Data Availability Statement

The datasets generated for this study are available on request to the corresponding author.

## Author Contributions

YW conceived and designed the study and coordination. SS, YM, MR, BH, and ZZ performed the simulations, optimized and drafted the protocol. YW wrote the paper. FC and FW reviewed and edited the manuscript. All authors read and approved the final manuscript.

## Funding

This work was supported by National Natural Science Foundation of China (31871537 to YW) and the Fundamental Research Funds for the Central Universities (2019QNA6019 to YW and 2019QNA6022 to FC).

## Conflict of Interest

The authors declare that the research was conducted in the absence of any commercial or financial relationships that could be construed as a potential conflict of interest.
